# Supplementary material for: Interaction Between PHF8 and a Segment of KDM2A, Which Is Controlled by the Phosphorylation Status at a Specific Serine in an Intrinsically Disordered Region of KDM2A, Regulates rRNA Transcription and Cell Proliferation in a Breast Cancer Cell Line
Source: Biomolecules. 2025 May 2;15(5):661. doi: 10.3390/biom15050661 (PMC12109296; doi:10.3390/biom15050661)
Supplement: Supplementary file 1 [file biomolecules-15-00661-s001.zip › biomolecules-3581530-supplementary S1-S9.pdf]

Supplementary Figures

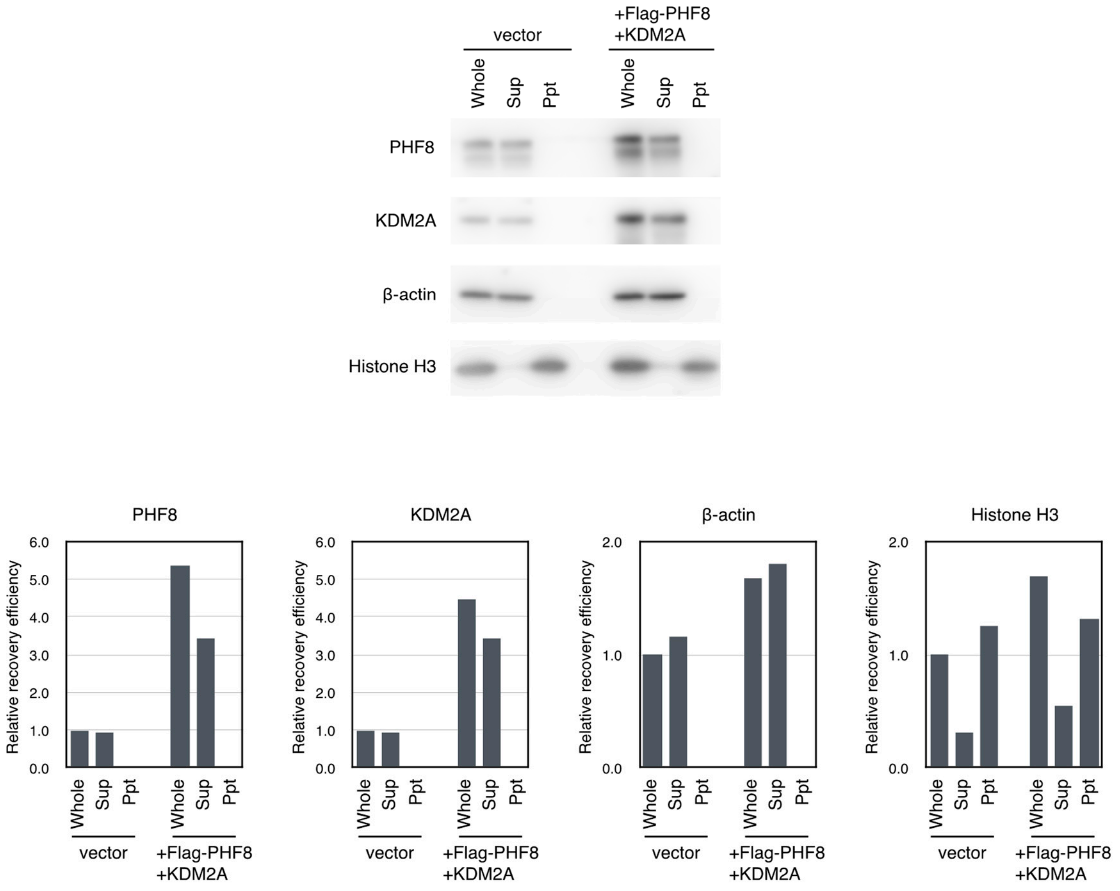

Figure S1. Extraction of KDM2A and PHF8 from cells using IP buffer. (A) 293T cells were transfected with empty vector or a vector expressing Flag-PHF8 and a vector expressing KDM2A by electroporation. Cells were cultured for 2 d, collected using trypsin and EDTA, and extracted with 3% SDS soln (Whole), or buffer for immunoprecipitation experiments (IP buffer). Proteins extracted with IP buffer were centrifuged at 20,000 g and separated into supernatant fraction (Sup) and precipitated fraction (Ppt). The 3% SDS soln were added to the collected samples, and samples prepared from the same number of cells were analyzed by western blotting using anti-PHF8 antibody, anti-KDM2A antibody (rabbit anti-KDM2A monoclonal antibody (Abcam, ab191387)), anti-β-actin antibody, and anti-histone H3 antibody (rabbit polyclonal anti-histone H3 antibody, Abcam, ab1791) (upper panels). Band intensities were measured and relative amounts were shown (lower panels).

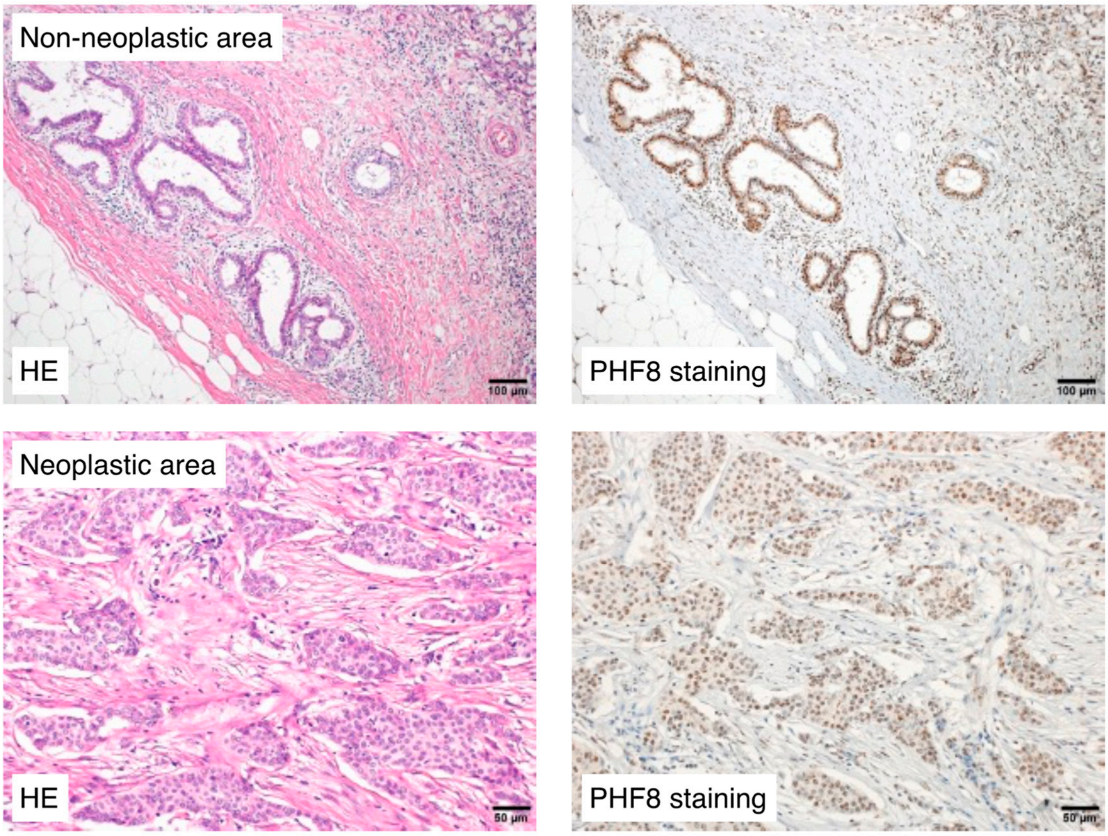

Figure S2. **PHF8 expression in breast cancer tissues.** Normal breast duct and breast tissue with scirrhous carcinoma (hematoxylin & eosin staining (HE), x100) (left hand side). A section of the tissues stained by anti-PHF8 antibody (Abcam ab84779) showing the expression of PHF8 in both neoplastic and non-neoplastic areas (x100). Positive staining is brown and counterstained nuclei are blue.

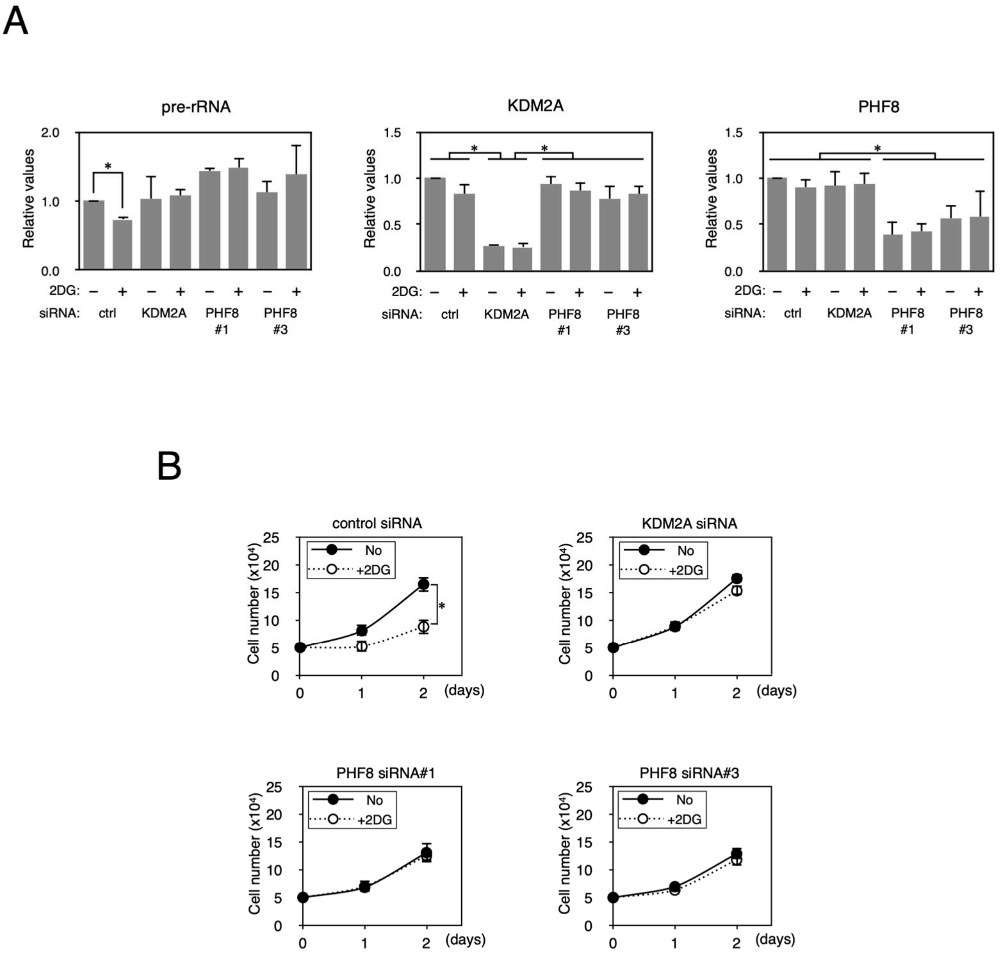

Figure S3. Effects of PHF8 and KDM2A knockdown on TNBC cell line MDA-MB-231. (A) MDA-MB-231 cells (European collection of authenticated cell cultures, ECACC, Catalogue No. 92020424) were transfected with stealth siRNAs for KDM2A or PHF8 (siPHF8#1 and siPHF8#3), and cultured for 3 d. The oligonucleotide sequence of stealth siRNA siPHF8#3 is 5'-CAUUCCACUUCAGUGUCCAUGUCCA-3'. Cells were replated in the growth medium. The next day, cells were cultured in the presence or absence of 3 mM 2DG for 2 h. Total RNA was isolated and pre-rRNA, KDM2A mRNA, and PHF8 mRNA were detected by RT-PCR. Results were normalized by Polr2a mRNA value. (B) Involvement of KDM2A and PHF8 in inhibition of cell proliferation by 2DG. Cells transfected as in (A) were replated and cultured in medium in the presence or absence of 3 mM 2DG. On the indicated days, cell numbers were counted. All experiments were performed three times, and mean values with standard deviations are indicated. *, $P < 0.05$.

A

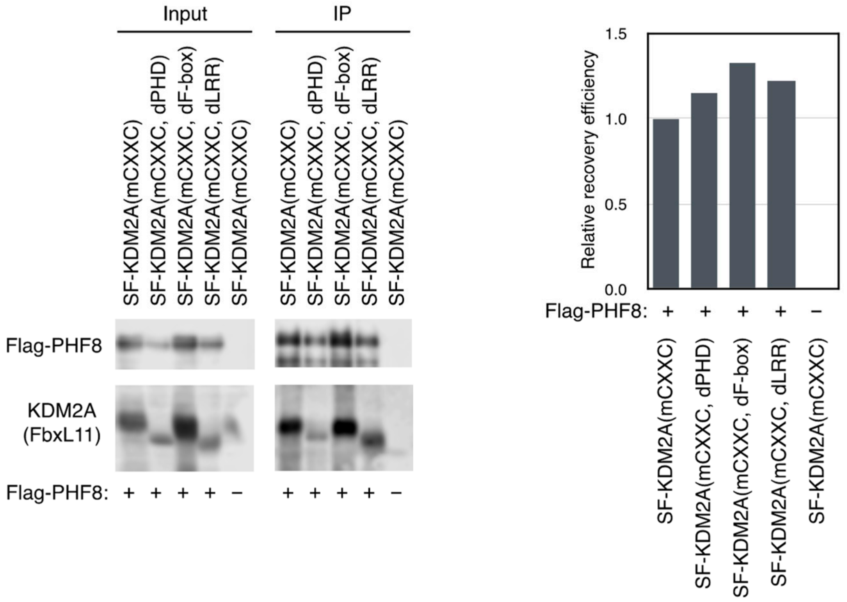
Input
IP
SF-KDM2A(mCXXC)
SF-KDM2A(mCXXC, dPHD)
SF-KDM2A(mCXXC, dF-box)
SF-KDM2A(mCXXC, dLRR)
SF-KDM2A(mCXXC)
Flag-PHF8
KDM2A
(FbxL11)
Flag-PHF8: + + + + −
Relative recovery efficiency
1.5
1.0
0.5
0.0
Flag-PHF8: + + + + −

B

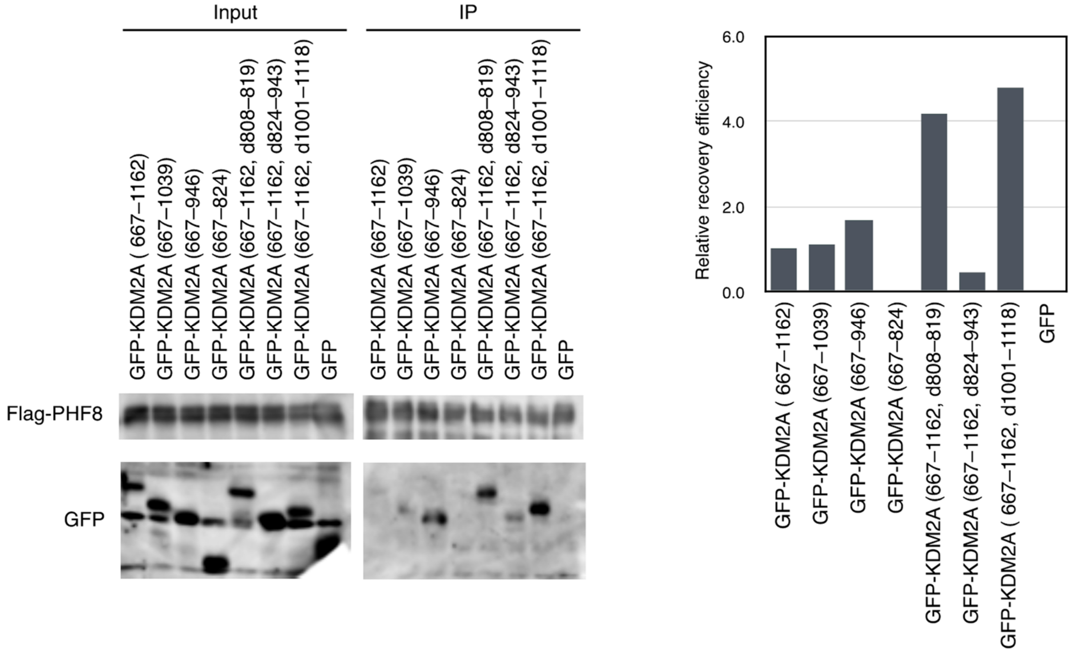
Input
IP
GFP-KDM2A (667–1162)
GFP-KDM2A (667–1039)
GFP-KDM2A (667–946)
GFP-KDM2A (667–824)
GFP-KDM2A (667–1162, d808–819)
GFP-KDM2A (667–1162, d824–943)
GFP-KDM2A (667–1162, d1001–1118)
GFP
Flag-PHF8
GFP
Relative recovery efficiency
6.0
4.0
2.0
0.0

C

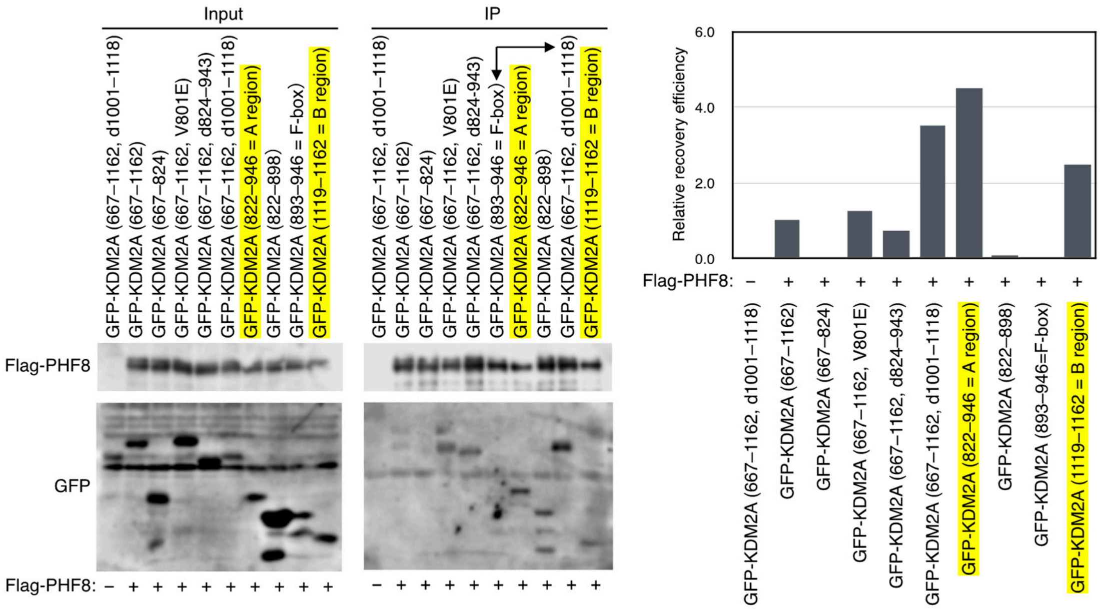

D

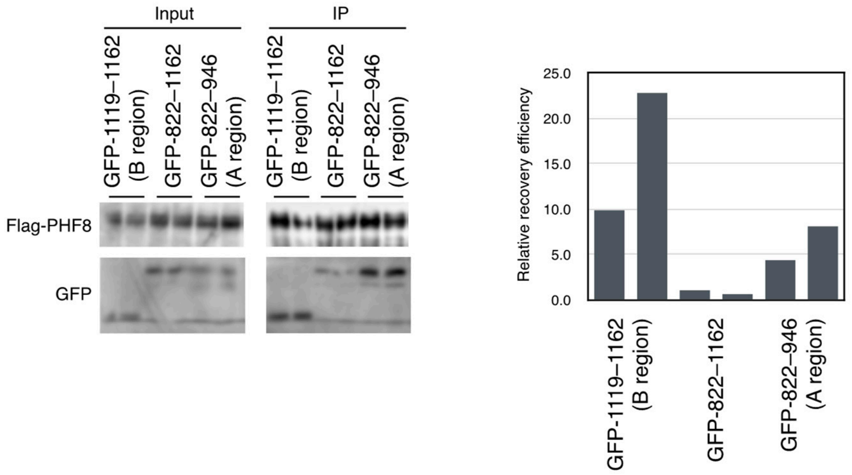

Figure S4 Identification of KDM2A regions that bind PHF8. (A) SF-KDM2A is encoded by the *KDM2A* gene and comprises amino acids 534–1162 of KDM2A [1,2]. Mutant SF-KDM2As, which were mutated in the domains as shown in the figure, were co-expressed with Flag-PHF8 into 293T cells. Cell lysates were immunoprecipitated with anti-Flag antibody-conjugated agarose and analyzed by Western blotting with anti-KDM2A (FbxL11) antibody (Abcam) and rabbit anti-Flag antibody (Sigma). Ten

percent of input samples were also analyzed for Flag-PHF8. Five percent of input samples were also analyzed for SF-KDM2A. The images for the immunoprecipitated samples were enhanced by long exposure. The relative recovery efficiency, SF-KDM2A (IP/Input)/FlagPHF8 (IP), was expressed as signals of immunoprecipitated SF-KDM2A against input of SF-KDM2A signals divided by immunoprecipitated Flag-PHF8 signals. (B-D) An expression vector encoding GFP fused with various fragments of KDM2A was cotransfected with a Flag-PHF8 expression vector into 293T cells by FuGENE6. Cell lysates were immunoprecipitated with anti-Flag antibody-conjugated agarose and analyzed by Western blotting with an anti-GFP antibody and a rabbit anti-Flag antibody (Sigma). Ten percent of input samples were also analyzed for Flag-PHF8. Five percent of input samples were also analyzed for GFP. GFP-fused KDM2A fragments that were co-expressed with Flag-PHF8 are shown in the Figure S2B, 2C, and 2D. The fragments of KDM2A used are summarized in Figure 2C. The relative recovery efficiency, GFP (IP/Input)/FlagPHF8 (IP), was expressed as signals of immunoprecipitated GFP against input GFP signals, which was divided by immunoprecipitated Flag-PHF8. (B) GFP-KDM2A (amino acids 667–1162) was deleted from the C-terminal end. The binding of GFP-KDM2A (amino acids 667–1039) to PHF8 was similar to that of GFP-KDM2A (amino acids 667–1162). The binding of GFP-KDM2A (amino acids 667–946) to PHF8 was stronger than that of GFP-KDM2A (amino acids 667–1162). GFP-KDM2A (amino acids 667–824) hardly bound to PHF8. The binding of GFP-KDM2A (amino acids 667–1162, d808–819) was stronger than GFP-KDM2A (amino acids 667–1162). KDM2A (amino acids 808–819) was close to the HP1γ binding site [3]. The binding of GFP-KDM2A (amino acids 667–1162, d824-943) to PHF8 was reduced compared to GFP-KDM2A (amino acids 667–1162). The binding of GFP-KDM2A (amino acids 667–1162, d1001–1118) was stronger than that of GFP-KDM2A (amino acids 667–1162). KDM2A (amino acids 1001–1118) contains leucine-rich repeats (LRR) (Figure 2C). GFP did not bind to PHF8. (C) The GFP-KDM2A (amino acids 667–1162, d1001–1118) that was not co-expressed with Flag-PHF8 was not collected by the Flag antibody in the first lane. The binding of GFP-KDM2A (amino acids 667–1162, V801E) to PHF8 was similar to that of GFP-KDM2A (amino acids 667–1162). The mutation at Val 801 was previously shown to inhibit the binding of KDM2A to the HP1γ binding site [3]. The binding of GFP-KDM2A (amino acids 667–1162, d824–943) was weaker than that of GFP-KDM2A (amino acids 667–1162). The binding of GFP-KDM2A (amino acids 667–1162, d1001–1118), with deleted LRR (Figure 2C), to PHF8 was stronger than that of GFP-KDM2A (amino acids 667–1162). The binding of GFP-KDM2A (amino acids 822–946) to PHF8 was stronger than that of GFP-KDM2A (amino acids 667–1162).

The GFP fusion protein KDM2A (amino acids 822–946) contained an F-box (Figure 2C). The GFP fusion protein KDM2A (amino acids 822–894) or KDM2A (amino acids 893–946 =F-box) scarcely bound to PHF8, suggesting that the existence of these two fragments in one peptide was required for PHF8 binding. The binding of GFP-KDM2A (amino acids 1119–1162 = B region) was stronger than that of GFP-KDM2A (amino acids 667–1162). (D) The bindings of GFP-KDM2A (amino acids 822–946 = A region) and GFP-KDM2A (amino acids 1119–1162 = B region) to PHF8 were stronger than that of GFP-KDM2A (amino acids 822–1162).

Together, these results suggest that two distinct regions of KDM2A (amino acids 822–946) and KDM2A (amino acids 1119–1162) are involved in the binding of KDM2A to PHF8.

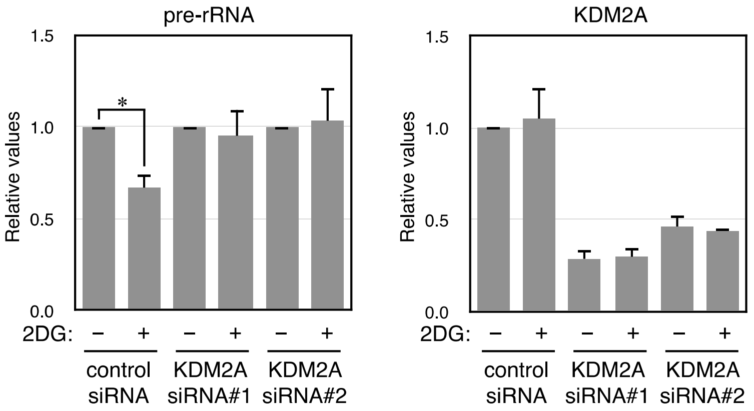

Figure S5 Reduction of rRNA transcription by mild starvation is dependent on KDM2A in 293T cells. 293T cells were transfected with siRNAs for KDM2A (siKDM2A#1 and siKDM2A#2) [1,2] for 3 d. Cells were replated and cultured for 1 d. The next day, cells were cultured in the presence or absence of 2 mM 2DG for 2 h, total RNA was isolated, and the pre-rRNA and KDM2A mRNA were detected by RT-PCR. The results were normalized by the values of Polr2a mRNA. The results show a KDM2A-dependent reduction of rRNA transcription by 2DG in 293T cells.

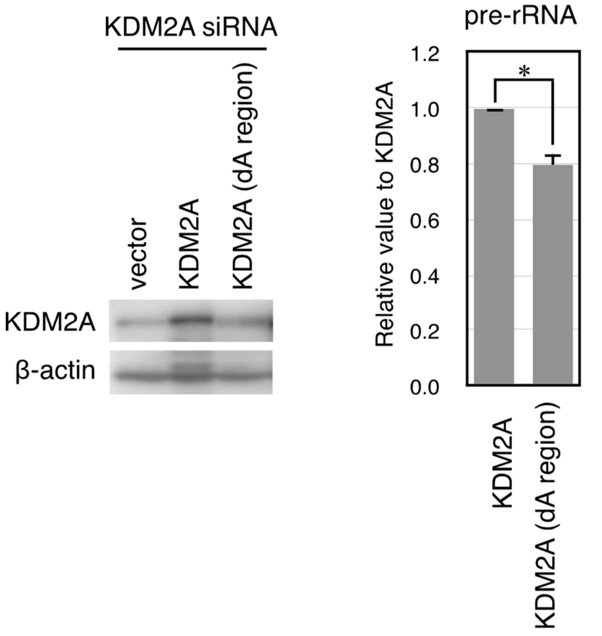

Figure S6. The A region is involved in reduction of rRNA transcription during mild starvation of breast cancer cell line MCF-7. After MCF-7 cells were transfected by an expression vector encoding KDM2A or KDM2A lacking the A region shown as KDM2A (dA region) by electroporation, cells were transfected with KDM2A siRNA (KDM2A), and cultured for 2 d. Cell lysates were analyzed by Western blotting using an anti-KDM2A antibody (Proteintech). β-actin was detected as a loading control (left panel). Total RNA was isolated from cells, and the levels of rRNA transcription were detected by RT-PCR. The results were normalized by the amounts of 28S rRNA (right panel). PCR primers for 28S rRNA amplification used are: Forward, 5′-ACCTGGCGCTAAACCATTCGT-3′ and Reverse, 5′-GGACAAACCCTTGTGTCGAGG-3′. P <0.005, for the difference of rRNA transcription between KDM2A (wild) and KDM2A (dA region). These results suggested that the A region is involved in the control of rRNA transcription in MCF-7 cells as well as 293T cells (Figure 3).

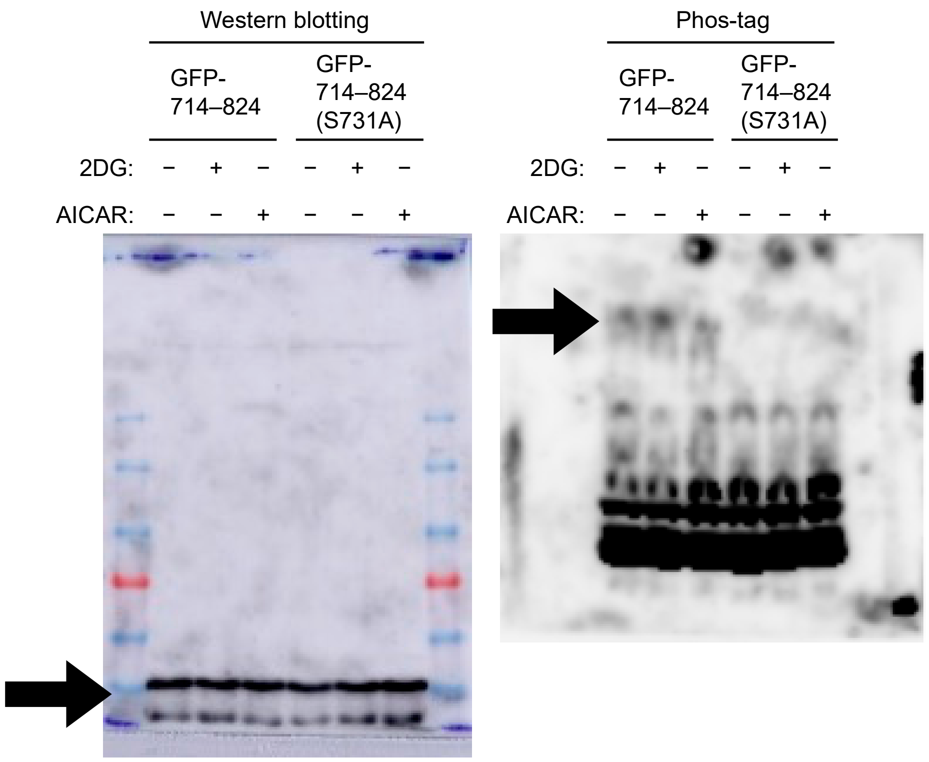

Figure S7. Detection of phosphorylation at Ser731 by phos-tag Western blotting. 293T cells were transfected with an expression vector encoding GFP-KDM2A (amino acids 714–824) and GFP-KDM2A (amino acids 714–824, S731A), by electroporation. Cells were lysed and analyzed by phos-tag Western blotting using a mouse monoclonal anti-GFP antibody (right-side figure). The shifted bands are indicated by arrows. Cell lysates were also analyzed by Western blotting to check the loaded proteins (left-side figure).

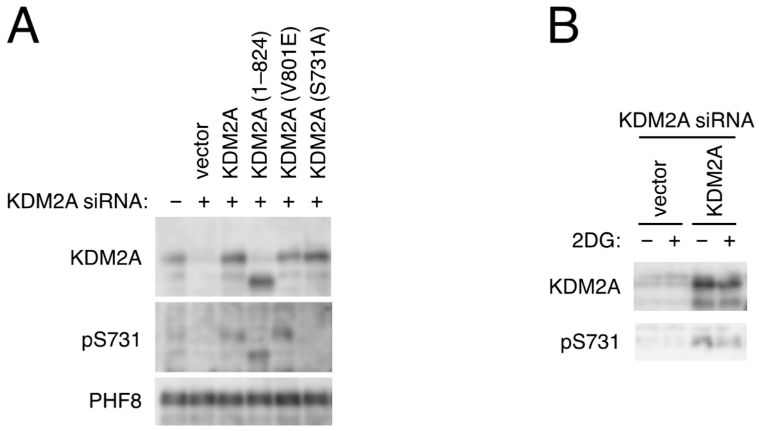

Figure S8. Antibody recognizing phosphorylated Ser731 of KDM2A. (A) Specific binding of the antibody to the phosphorylated Ser731 of KDM2A. After MCF-7 cells were transfected with siRNA for KDM2A or control siRNA for 2 d, an expression vector encoding KDM2A, KDM2A (amino acids 1–824), KDM2A mutant (V801E) that had lost binding for HP1γ [3], or KDM2A mutant (S731A), was transfected by electroporation. Cells were lysed and analyzed by Western blotting using an anti-KDM2A antibody (Proteintech) and the anti-phosphorylated Ser731 antibody. The results showed that the antibody specifically recognized phosphorylated Ser731. (B) The phosphorylation level of KDM2A was reduced by 2DG in 293T cells. After 293T cells were transfected with siRNA for KDM2A for 2 d, an expression vector encoding KDM2A was transfected by electroporation, and cultured for 2 d. After cells were treated with 2 mM 2DG for 2 h, cell lysates were analyzed by Western blotting as in A. The 2DG treatment for 2 h reduced the signal of phosphorylated Ser731.

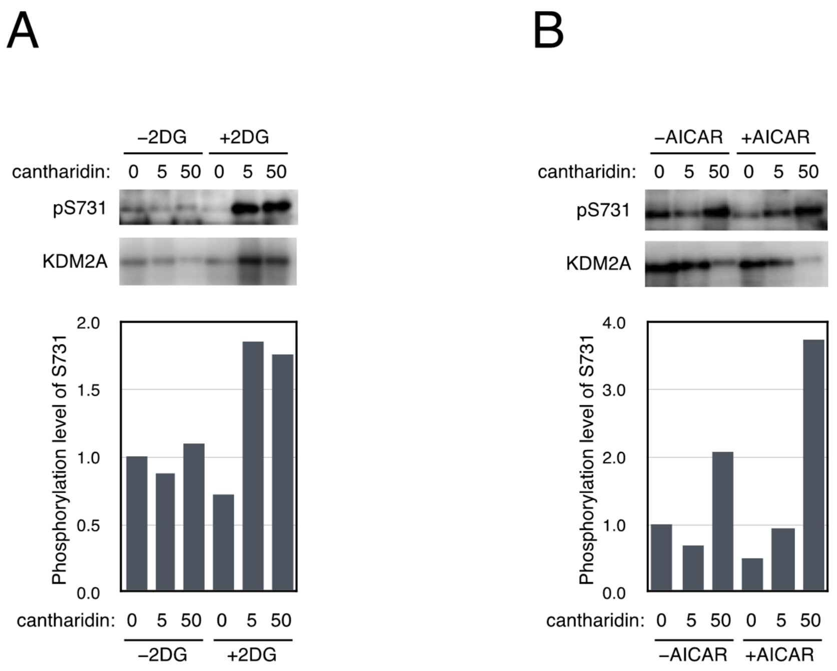

Figure S9. Evidence for the involvement of a phosphatase in the dephosphorylation of Ser731 in KDM2A during starvation. An expression vector encoding KDM2A was transfected into 293T cells by electroporation, and then siRNA for KDM2A was transfected. After cultured for 2 d, cells were treated with a phosphatase inhibitor, cantharidin, at the indicated concentrations, for 4 h in the presence or absence of 2mM 2DG (A) or 0.5 mM AICAR (B). Cells were collected and analyzed by Western blotting using anti-phosphorylated Ser731 antibody and anti-KDM2A antibody (Abcam, ab191387) (upper panel). Relative phosphorylation levels were expressed as the value of phosphorylated Ser731 divided by the value of KDM2A. The value without 2DG (A), AICAR (B), and cantharidin, was expressed as 1 (lower panel).

References

1. Tanaka, Y.; Yano, H.; Ogasawara, S.; Yoshioka, S.; Imamura, H.; Okamoto, K.; Tsuneoka, M. Mild Glucose Starvation Induces KDM2A-Mediated H3K36me2 Demethylation through AMPK To Reduce rRNA Transcription and Cell Proliferation. *Mol Cell Biol* **2015**, *35*, 4170-4184, doi:10.1128/MCB.00579-15.
2. Tanaka, Y.; Okamoto, K.; Teye, K.; Umata, T.; Yamagiwa, N.; Suto, Y.; Zhang, Y.; Tsuneoka, M. JmjC enzyme KDM2A is a regulator of rRNA transcription in response to starvation. *EMBO J* **2010**, *29*, 1510-1522, doi:10.1038/emboj.2010.56.
3. Okamoto, K.; Tanaka, Y.; Ogasawara, S.; Obuse, C.; Nakayama, J.I.; Yano, H.; Tsuneoka, M. KDM2A-dependent reduction of rRNA transcription on glucose starvation requires HP1 in cells, including triple-negative breast cancer cells. *Oncotarget* **2019**, *10*, 4743-4760, doi:10.18632/oncotarget.27092.
