# Supplementary material for: Interaction Between PHF8 and a Segment of KDM2A, Which Is Controlled by the Phosphorylation Status at a Specific Serine in an Intrinsically Disordered Region of KDM2A, Regulates rRNA Transcription and Cell Proliferation in a Breast Cancer Cell Line
Source: Biomolecules. 2025 May 2;15(5):661. doi: 10.3390/biom15050661 (PMC12109296; doi:10.3390/biom15050661)
Supplement: Supplementary file 1 [file biomolecules-15-00661-s001.zip › biomolecules-3581530-supplementary S10 original WB.pdf]

Figure 1

A

	Input		
Flag-PHF8	-	+	+
KDM2A	+	-	+

	IP		
Flag-PHF8	-	+	+
KDM2A	+	-	+

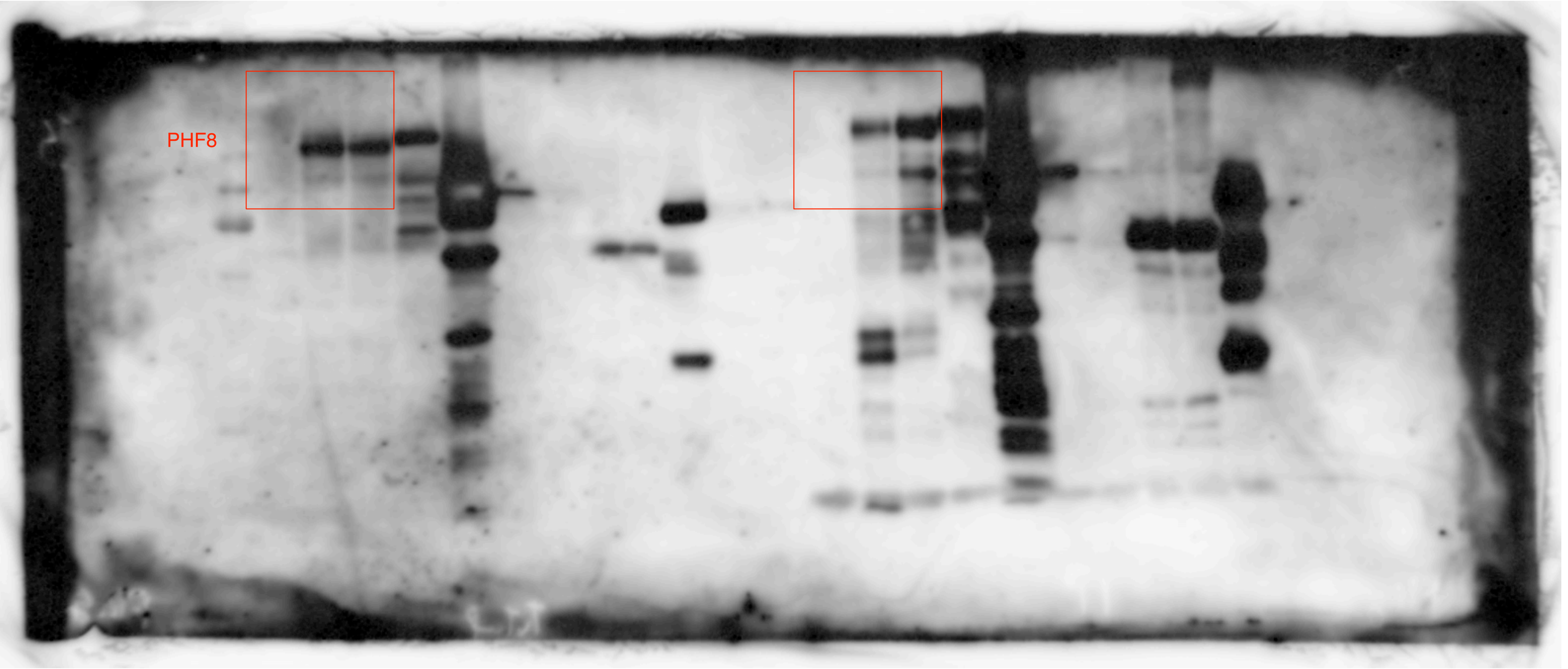

	Input		
Flag-PHF8	-	+	+
KDM2A	+	-	+

IP		
-	+	+
+	-	+

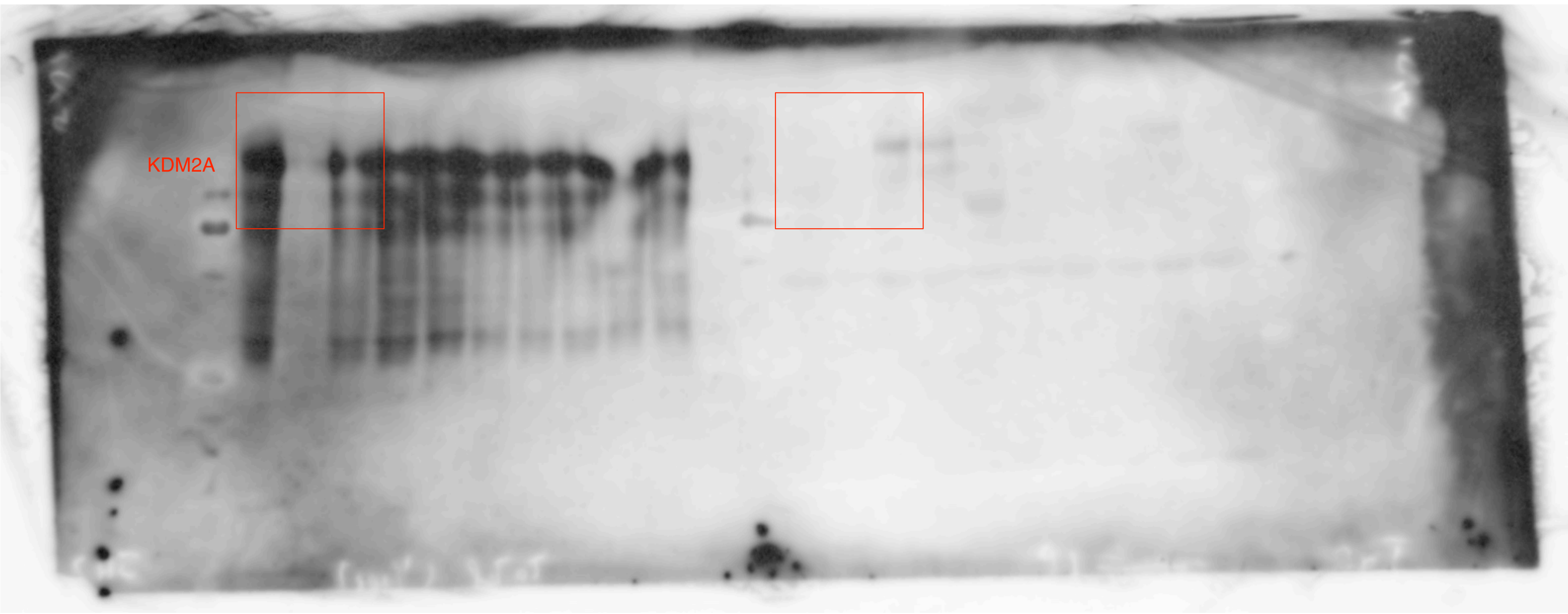

B

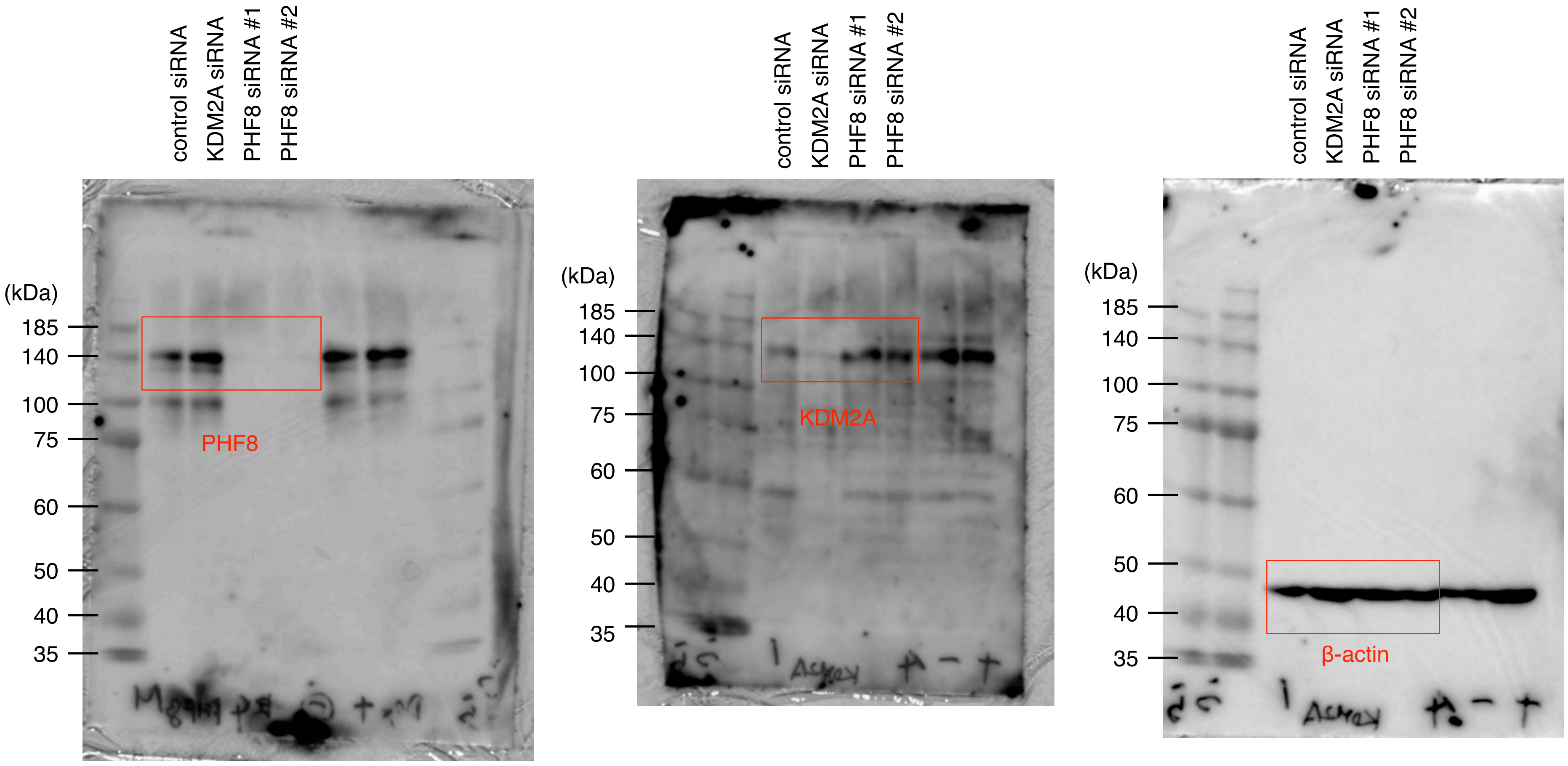
control siRNA
KDM2A siRNA
PHF8 siRNA #1
PHF8 siRNA #2
(kDa)
185
140
100
75
60
50
40
35
PHF8
control siRNA
KDM2A siRNA
PHF8 siRNA #1
PHF8 siRNA #2
(kDa)
185
140
100
75
60
50
40
35
KDM2A
control siRNA
KDM2A siRNA
PHF8 siRNA #1
PHF8 siRNA #2
(kDa)
185
140
100
75
60
50
40
35
β-actin

Figure 2

A

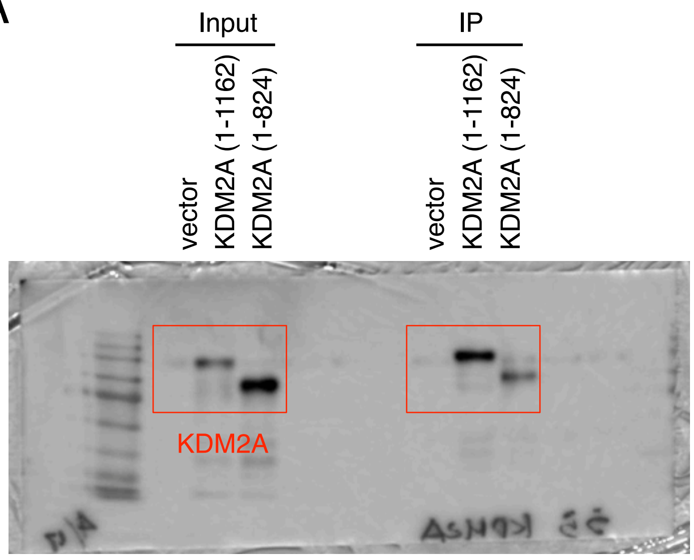

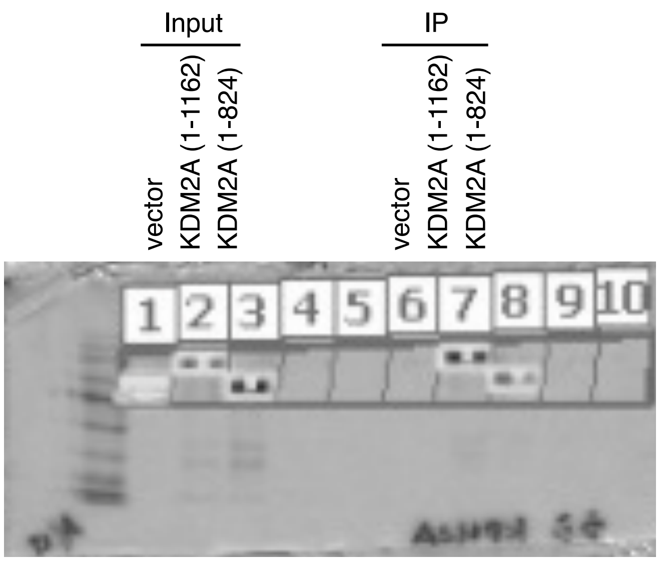

Lane	Volume
1	ND
2	121137
3	383682
6	ND
7	290692
8	139342

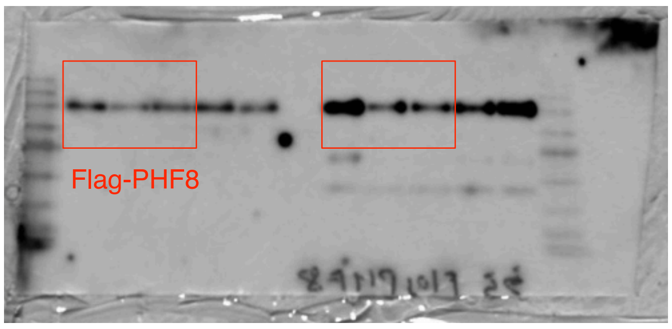

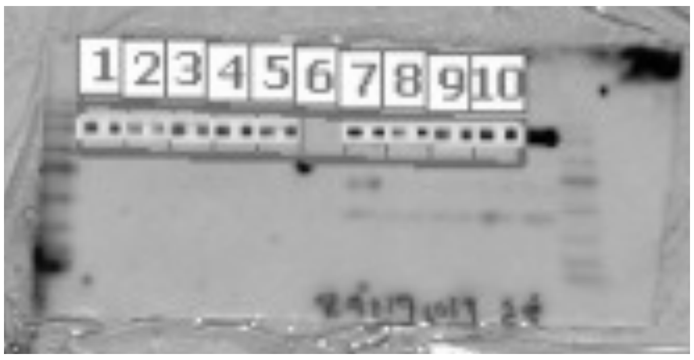

Lane	Volume
1	67427
2	37477
3	58689
7	258128
8	84103
9	75561

B

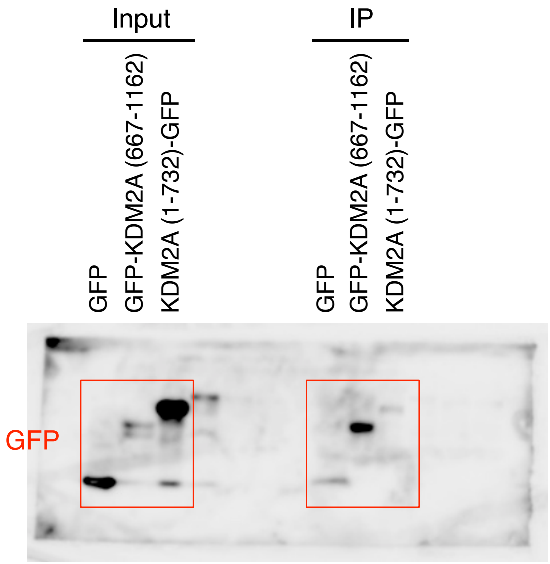

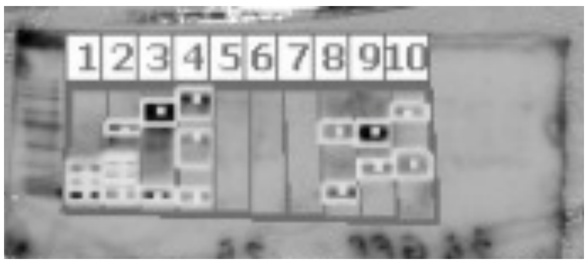

Lane	Volume
1	137141
2	54932
3	511615
8	39028
9	143271
10	14462

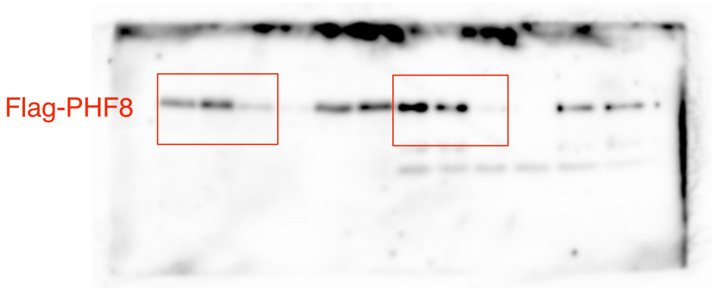

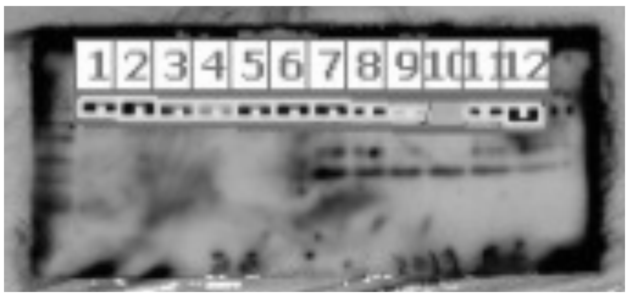

Lane	Volume
1	187771
2	311089
3	104505
7	382588
8	249790
9	18737

D

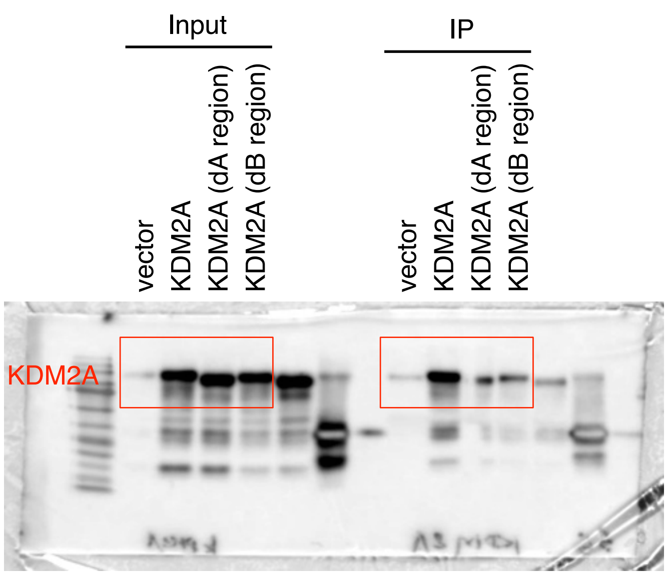

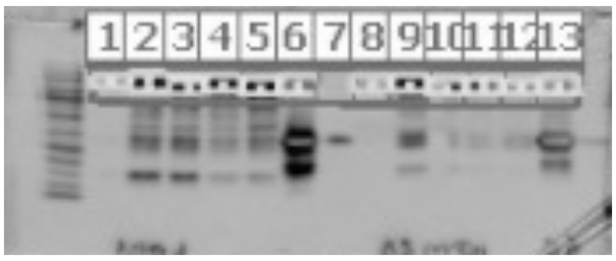

Lane	Volume
1	46284
2	352390
3	420534
4	330479
8	32784
9	335657
10	102698
11	111625

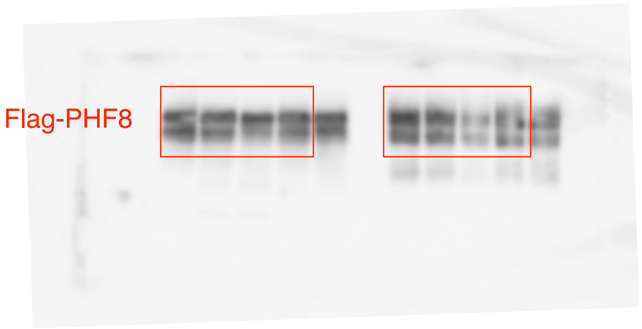

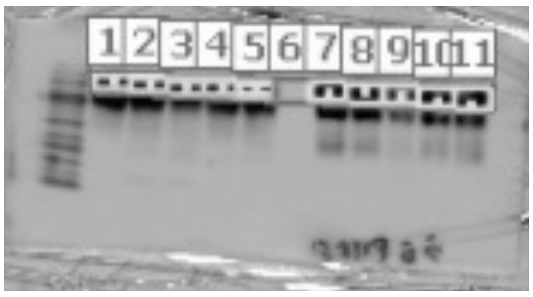

Lane	Volume
1	228203
2	299512
3	340053
4	288566
7	290896
8	242176
9	124960
10	135990

Figure 3

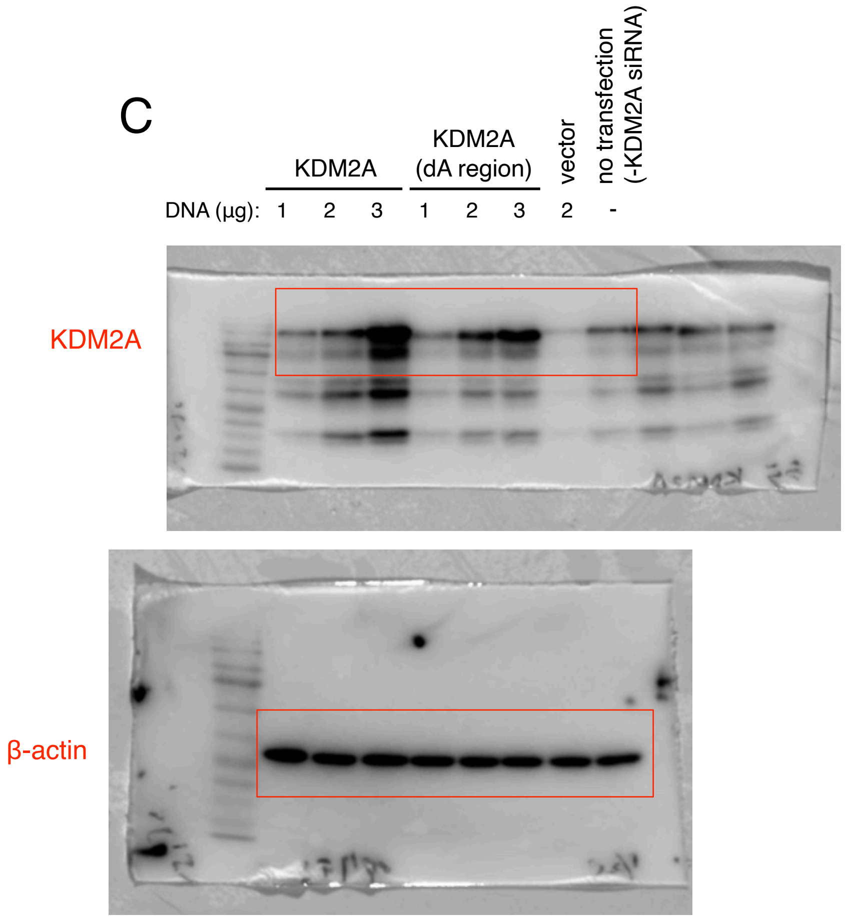
C
KDM2A
KDM2A (dA region)
vector
no transfection (-KDM2A siRNA)
DNA (µg): 1 2 3 1 2 3 2 -
KDM2A
β-actin

Figure 4

A

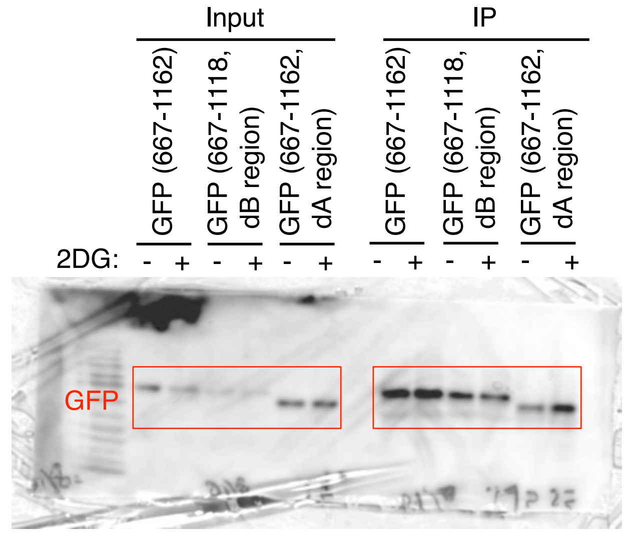

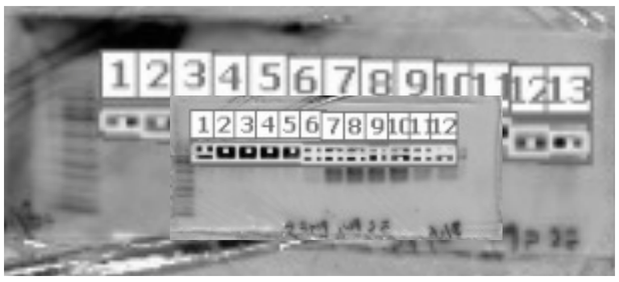

Lane	Volume
1	82788
2	71914
3	43493
4	53349
5	102018
6	123294
8	206067
9	273100
10	183406
11	151491
12	176438
13	205415

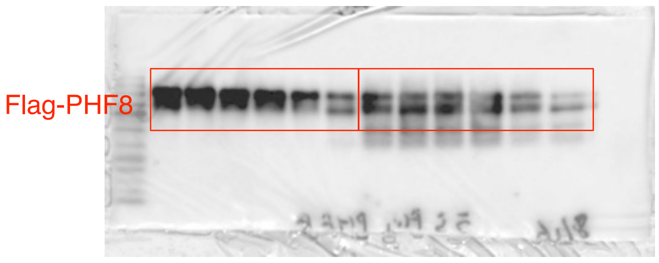

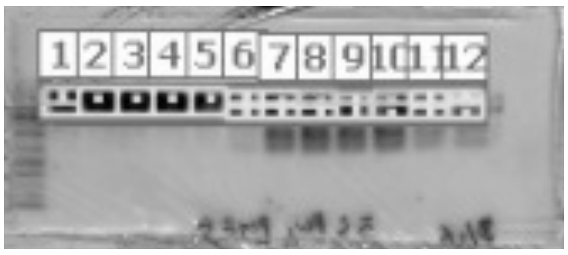

Lane	Volume
1	456332
2	564755
3	478071
4	474209
5	373311
6	316491
7	191359
8	195378
9	191593
10	210744
11	247156
12	165152

C

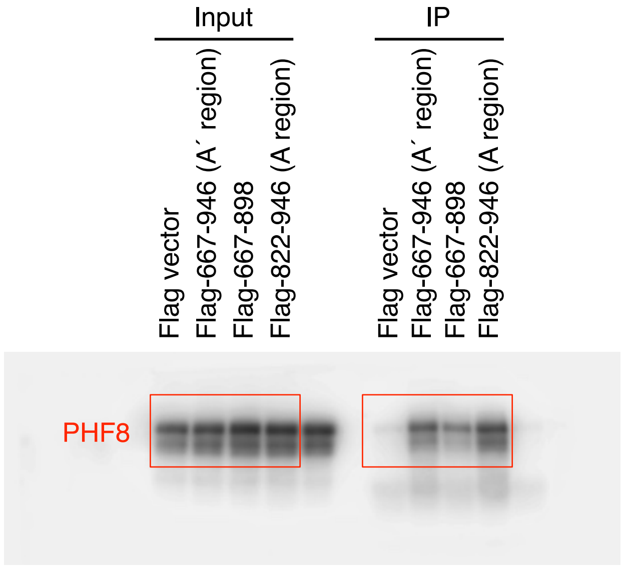

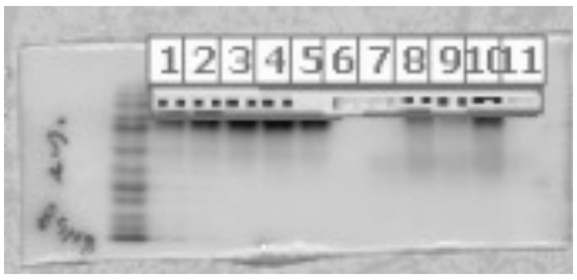

Lane	Volume
1	290649
2	346569
3	407883
4	416918
7	29208
8	185380
9	149123
10	238866

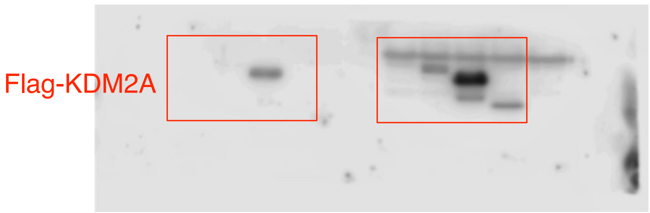

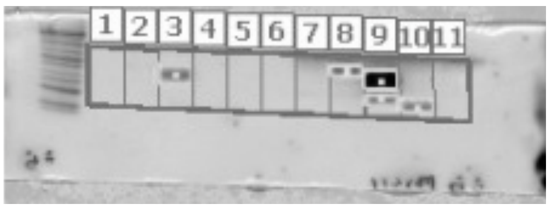

Lane	Volume
1	ND
2	ND
3	132736
4	ND
7	ND
8	51865
9	643466
10	81976

D

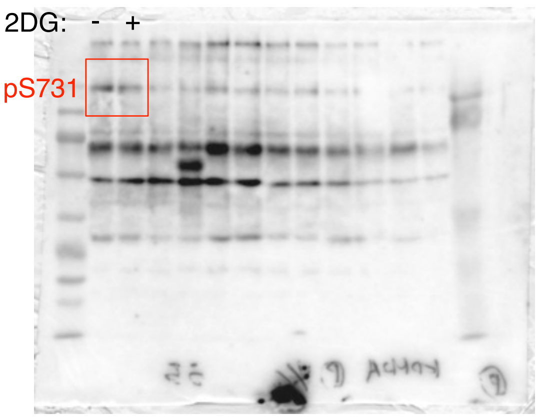

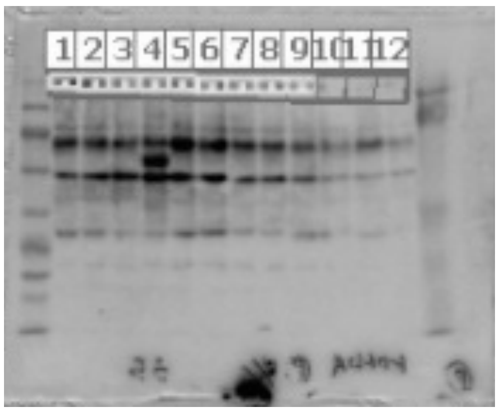

Lane	Volume
1	20389
2	17703

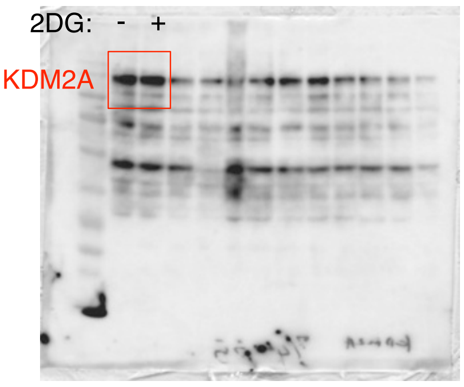

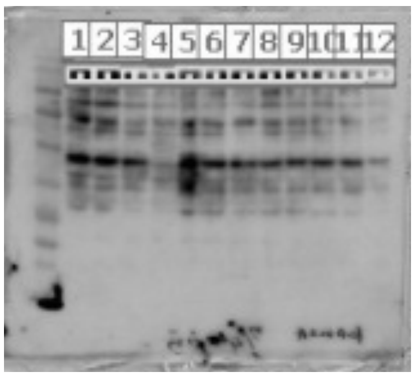

Lane	Volume
1	134543
2	152817

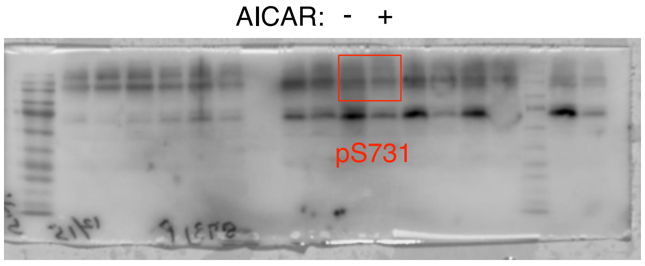

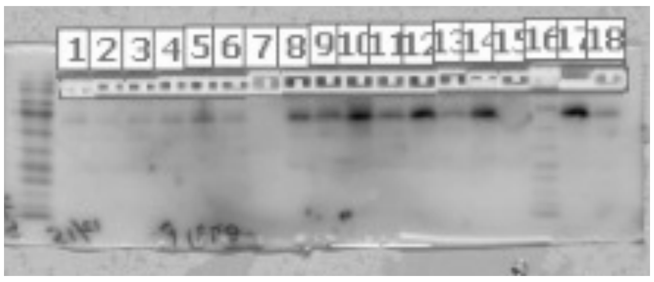

Lane	Volume
10	64992
11	71328

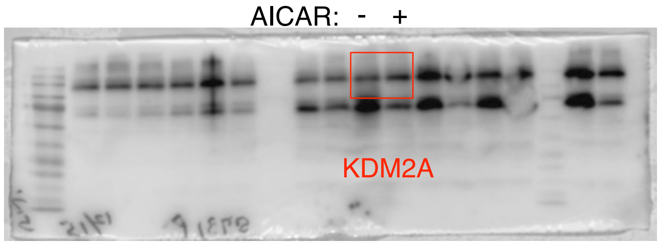

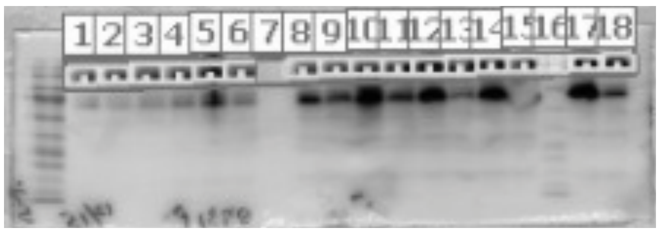

Lane	Volume
10	83770
11	132886

Figure 5

A

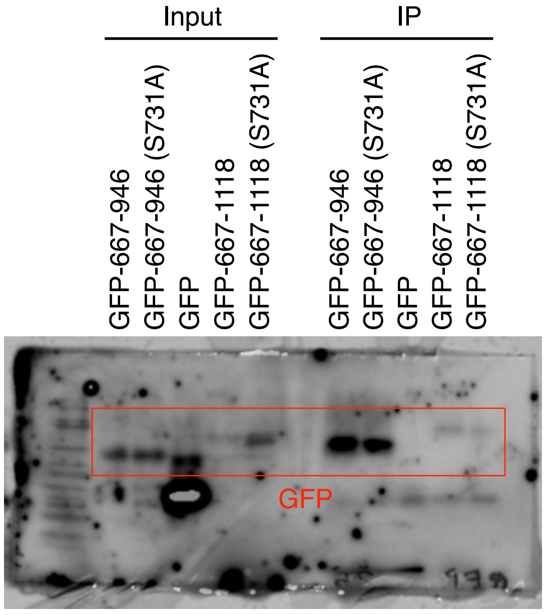

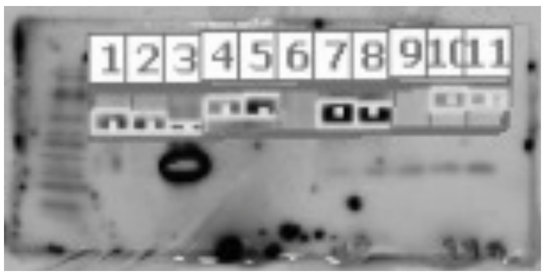

Lane	Volume
1	29256
2	25012
3	8175
4	13171
5	33164
7	94669
8	60093
9	ND
10	11369
11	6431

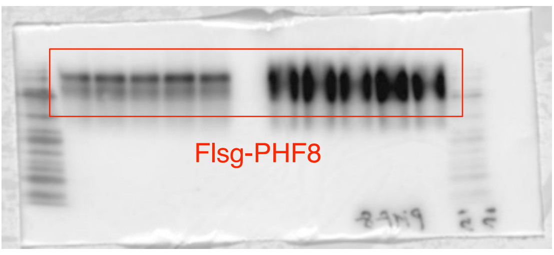

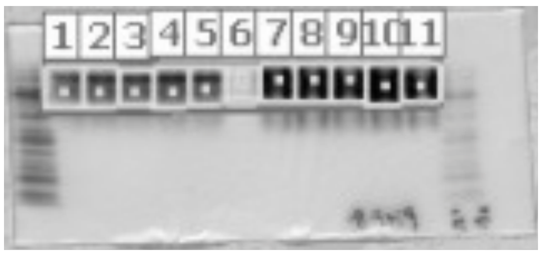

Lane	Volume
1	290305
2	484687
3	435507
4	486363
5	432887
7	628594
8	588235
9	578101
10	782364
11	620465

B

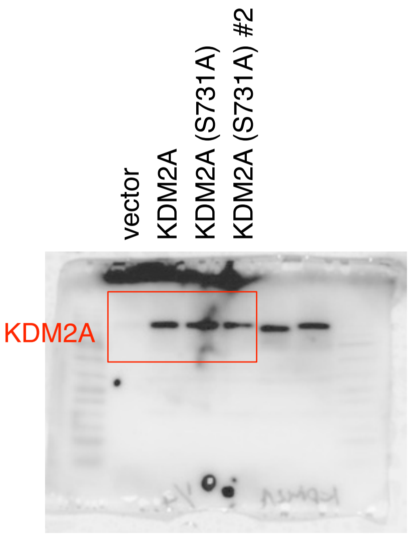

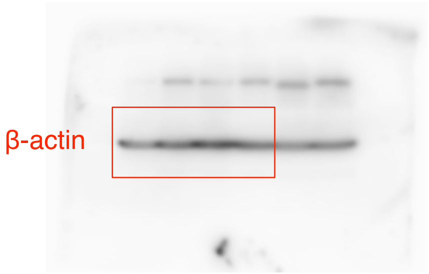

Figure S2

A

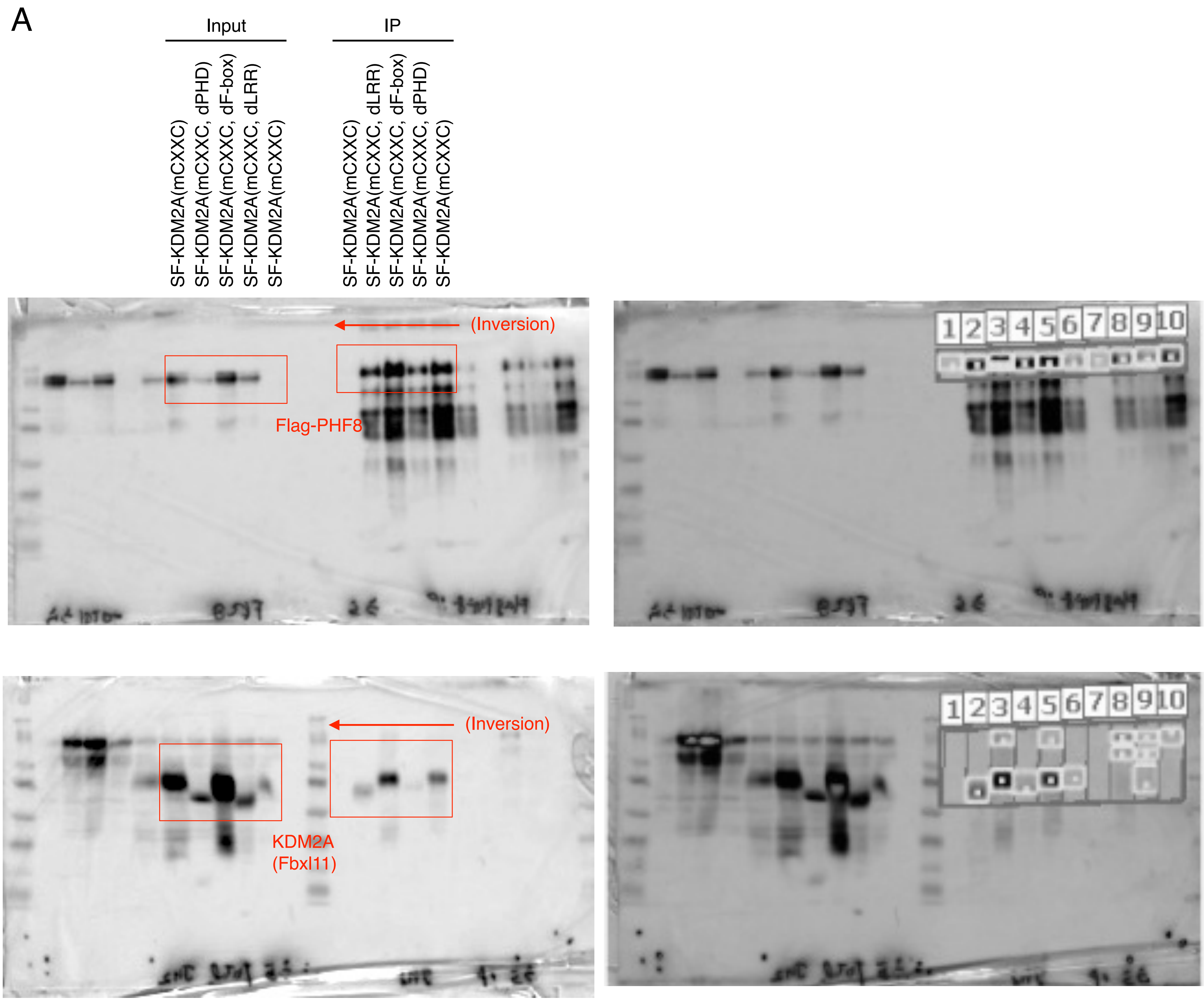

Lane	Volume
5	471567
4	248173
3	683425
2	292638
1	6313

Lane	Volume
5	914077
4	198368
3	1243141
2	576883
1	ND

Figure S2

B

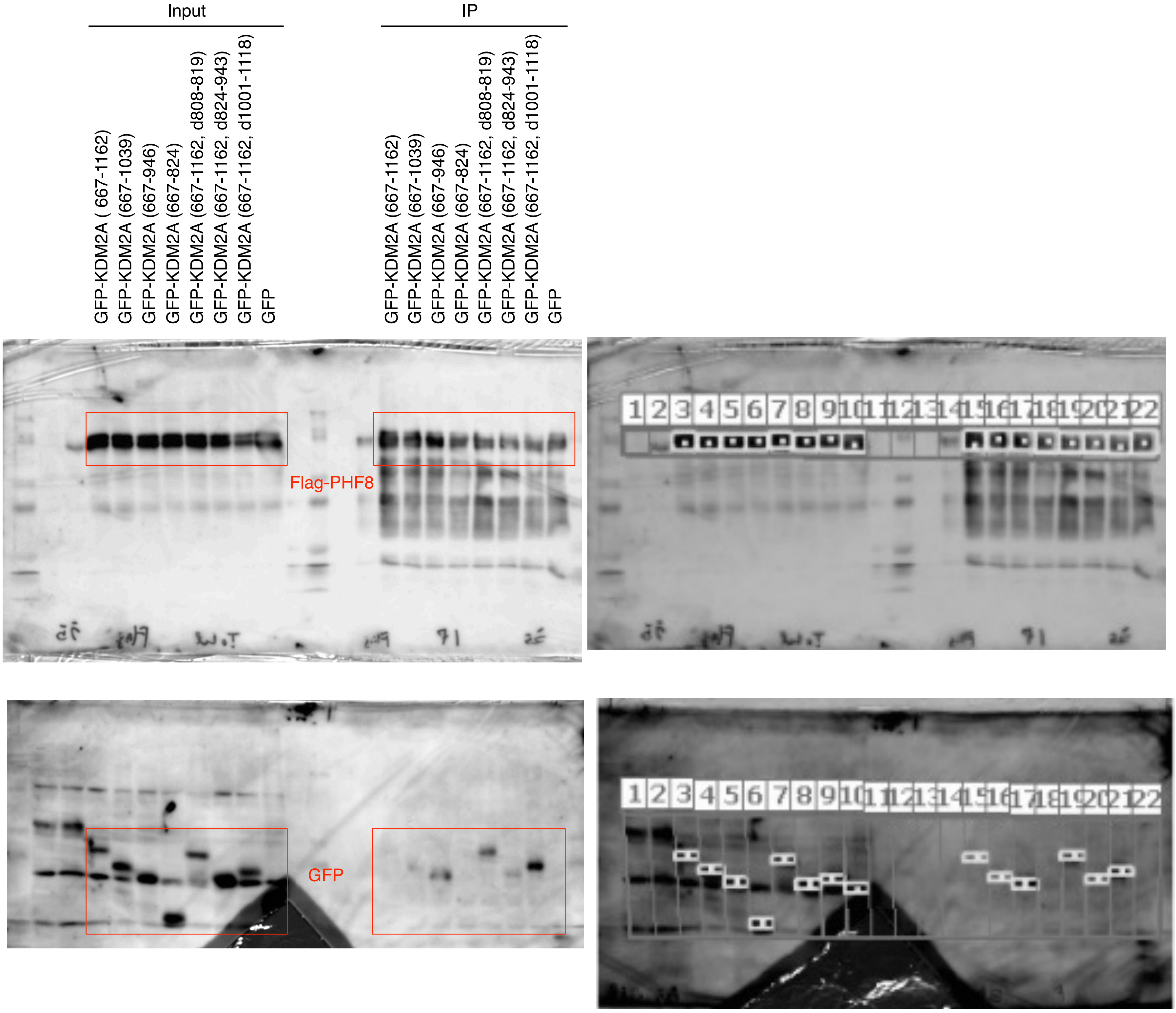

Lane	Volume
15	266180
16	290093
17	378099
18	283486
19	294676
20	253910
21	263810
22	289995

Lane	Volume
15	9142
16	15000
17	34325
18	ND
19	27129
20	14511
21	47241
22	ND

C

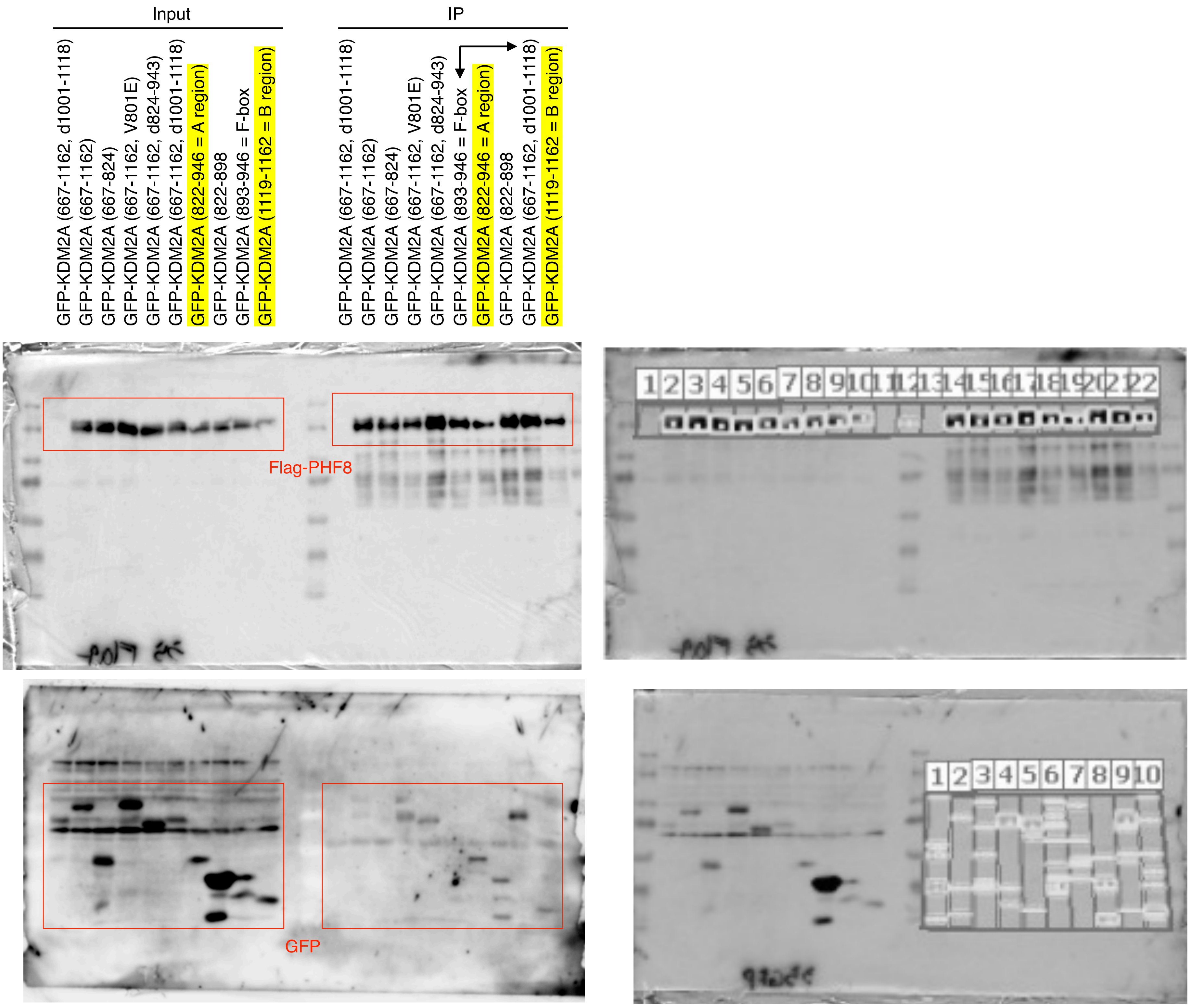

Lane	Volume
13	ND
14	440959
15	416547
16	365389
17	726018
21	628963
19	288026
20	618032
18	483365
22	341775

Lane	Volume
1	ND
2	5156
3	ND
4	7407
5	6325
9	10683
7	7380
8	5872
6	ND
10	5059

D

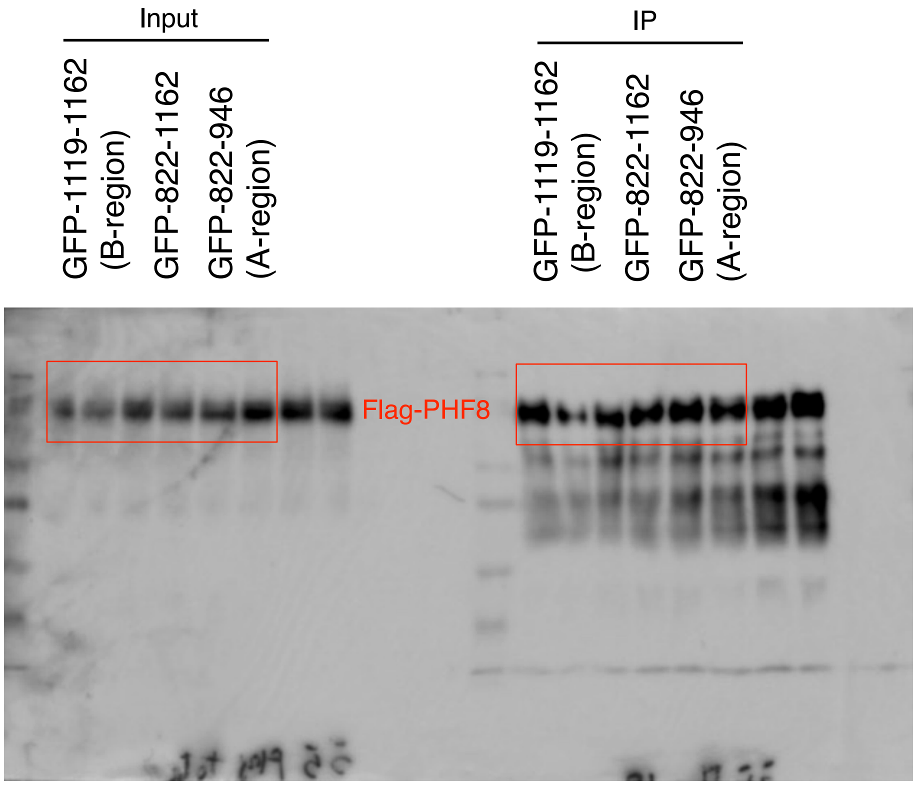

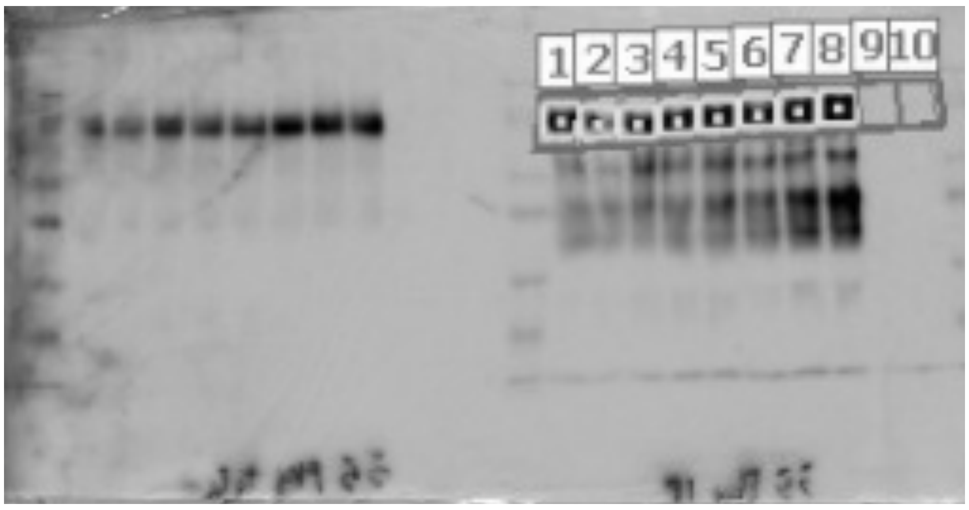

Lane	Volume
1	444429
2	236311
3	378179
4	468243
5	544638
6	454119

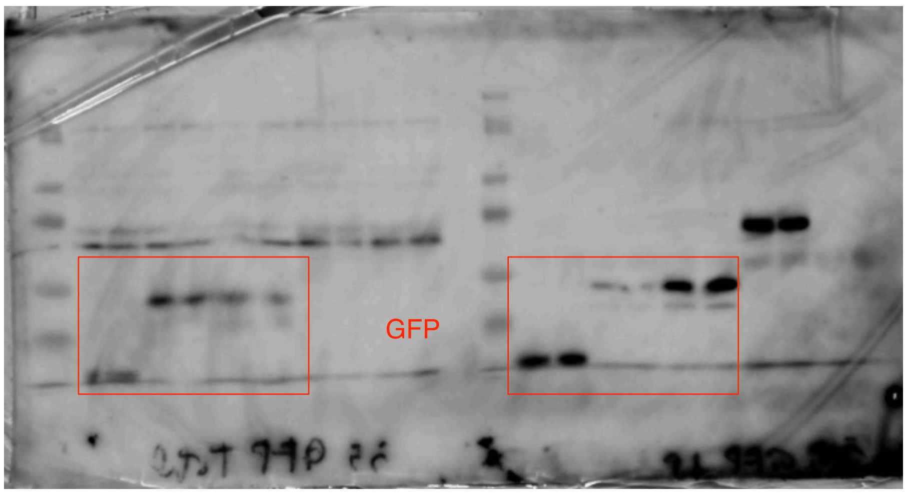

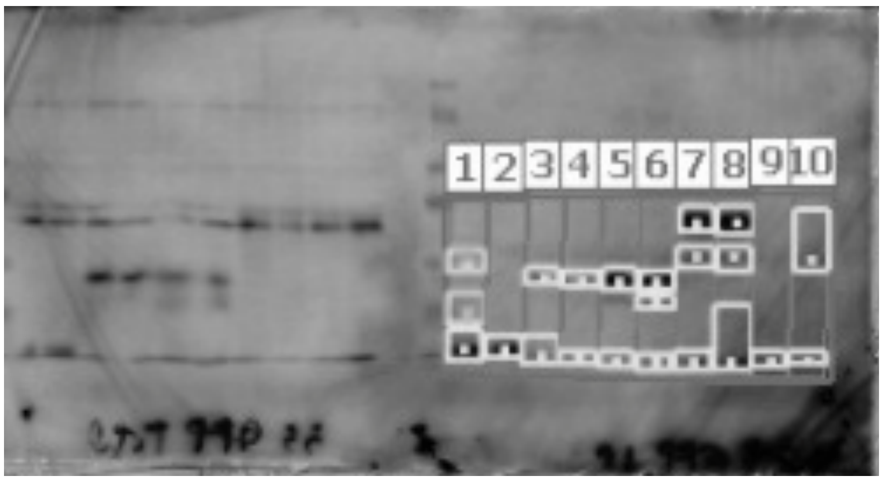

Lane	Volume
1	385357
2	320637
3	80144
4	51443
5	337641
6	393190

Figure S4

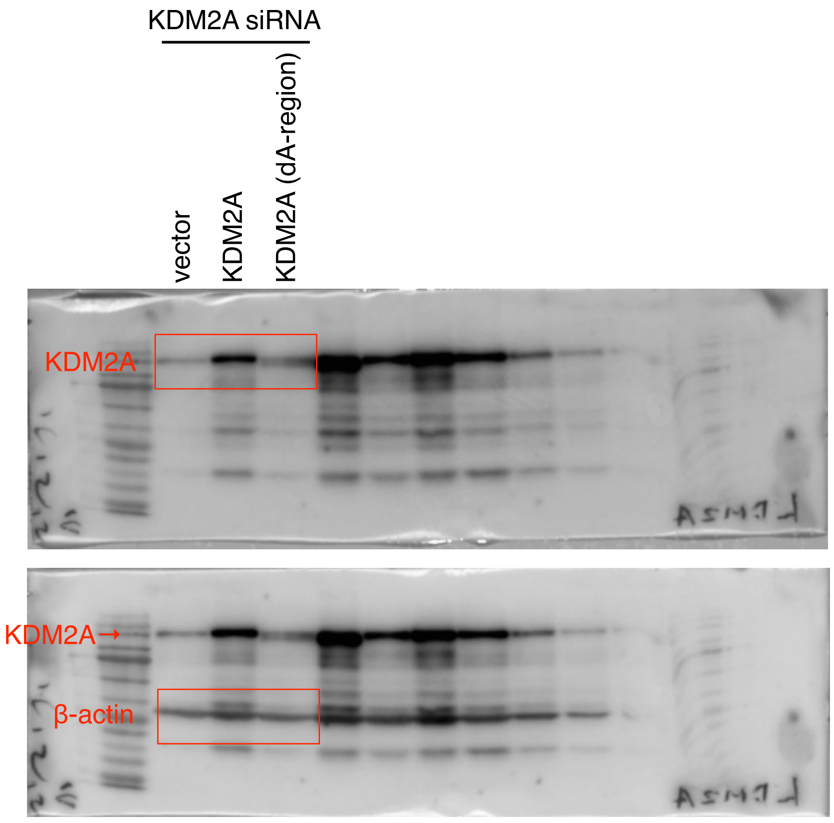

KDM2A siRNA
vector
KDM2A
KDM2A (dA-region)
KDM2A
KDM2A→
β-actin

Figure S6

A

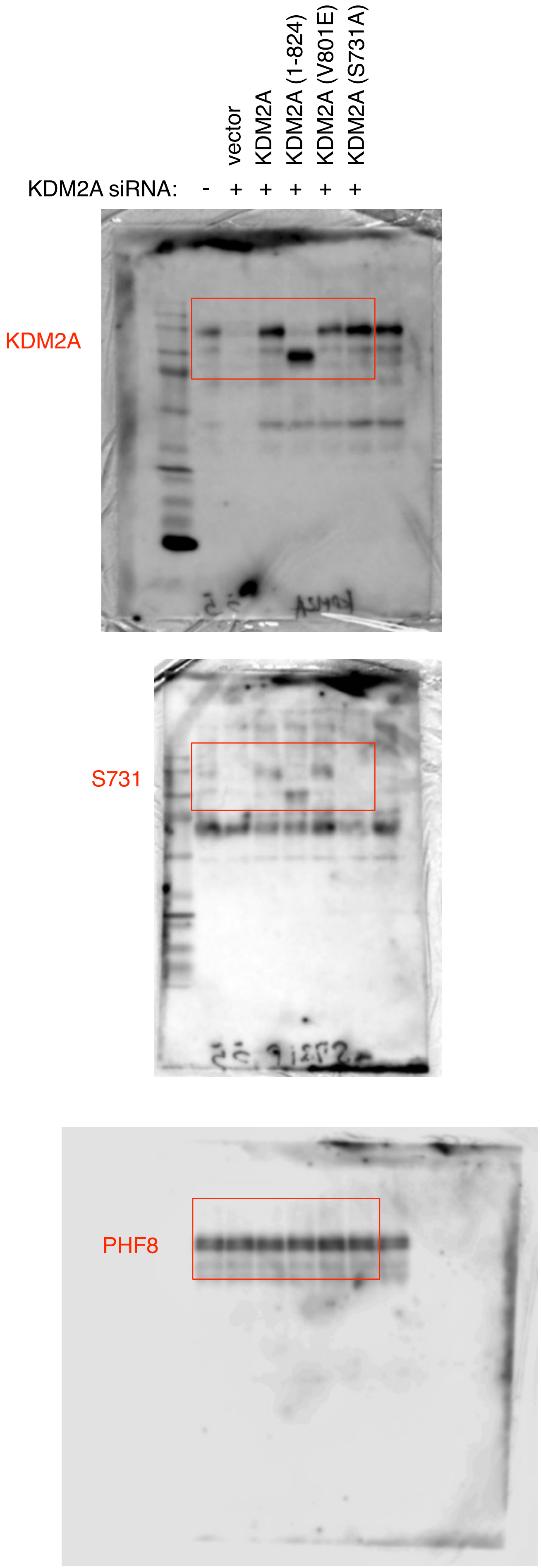

vector
KDM2A
KDM2A (1-824)
KDM2A (V801E)
KDM2A (S731A)
KDM2A siRNA: - + + + + +
KDM2A
S731
PHF8

B

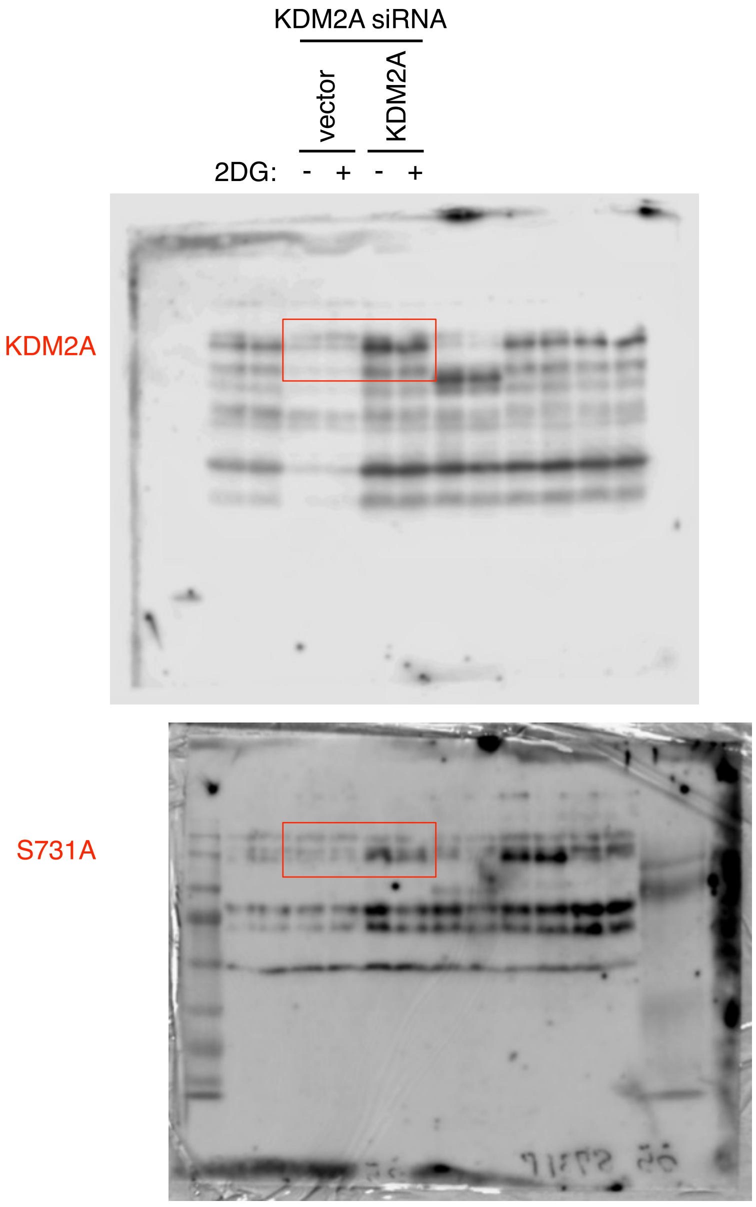

KDM2A siRNA
vector
KDM2A
2DG: - + - +
KDM2A
S731A
